# Prevalent *emm* Types among Invasive GAS in Europe and North America since Year 2000

**DOI:** 10.3389/fpubh.2018.00059

**Published:** 2018-03-09

**Authors:** Giovanni Gherardi, Luca Agostino Vitali, Roberta Creti

**Affiliations:** ^1^Microbiology Unit, Department of Medicine, Campus Bio-Medico University, Rome, Italy; ^2^Microbiology Unit, School of Pharmacy, University of Camerino, Camerino, Italy; ^3^Department of Infectious Diseases, Istituto Superiore di Sanità, Rome, Italy

**Keywords:** group A streptococcus invasive disease, *Streptococcus pyogenes*, *emm* types, Europe, North America, group A streptococci

## Abstract

**Background:**

*Streptococcus pyogenes* or group A streptococcus (GAS) is an important human pathogen responsible for a broad range of infections, from uncomplicated to more severe and invasive diseases with high mortality and morbidity. Epidemiological surveillance has been crucial to detect changes in the geographical and temporal variation of the disease pattern; for this purpose the M protein gene (*emm)* gene typing is the most widely used genotyping method, with more than 200 *emm* types recognized. Molecular epidemiological data have been also used for the development of GAS M protein-based vaccines.

**Methods:**

The aim of this paper was to provide an updated scenario of the most prevalent GAS *emm* types responsible for invasive infections in developed countries as Europe and North America (US and Canada), from 1st January 2000 to 31st May 2017. The search, performed in PubMed by the combined use of the terms (“*emm*”) and (“invasive”) retrieved 264 articles, of which 38 articles (31 from Europe and 7 from North America) met the inclusion criteria and were selected for this study. Additional five papers cited in the European articles but not retrieved by the search were included.

**Results:**

*emm*1 represented the dominant type in both Europe and North America, replaced by other *emm* types in only few occasions. The seven major *emm* types identified (*emm*1, *emm*28, *emm*89, *emm*3, *emm*12, *emm*4, and *emm*6) accounted for approximately 50–70% of the total isolates; less common *emm* types accounted for the remaining 30–50% of the cases. Most of the common *emm* types are included in either one or both the 26-valent and 30-valent vaccines, though some well-represented *emm* types found in Europe are not.

**Conclusion:**

This study provided a picture of the prevalent *emm* types among invasive GAS (iGAS) in Europe and North America since the year 2000 onward. Continuous surveillance on the *emm*-type distribution among iGAS infections is strongly encouraged also to determine the potential coverage of the developing multivalent vaccines.

## Introduction

*Streptococcus pyogenes* or group A streptococcus (GAS) is a human Gram-positive bacterial species that either can exist as commensal or can be responsible for a broad range of infections, ranging from uncomplicated throat and skin infections to more severe and invasive diseases, such as bacteremia, soft tissue infections, necrotizing fasciitis, and septic shock; it represents, therefore, on a global scale, an important cause of morbidity and mortality (1). In addition, symptomatic infections can be involved in alterations of the immune system leading to post-streptococcal autoimmune sequelae, such as acute rheumatic fever (ARF) and chronic rheumatic heart disease (RHD), and post-streptococcal glomerulonephritis. ARF/RHD is an uncommon disease today in most high-income countries but it remains the major cause of acquired heart disease in adolescents and young adults in the developing world, responsible for at least 350,000 premature deaths per year (2).

Due to the size and clinical severity of the GAS disease burden, epidemiological surveillance has been crucial to detect changes in the disease pattern in various populations. Typing of collected bacterial isolates has been an important part of the epidemiological surveillance of GAS disease. Several different methods have been described and are available for GAS typing (3, 4). Classical serotyping methods based on the different forms of M, T, and OF surface antigens have been replaced by sequence typing of the N-terminal part of the M protein (*emm*) gene in the late 1990s (4–6), and it is now the most widely used genotyping method for GAS. So far, more than 200 *emm* types have been identified (http://www.cdc.gov/streplab/MProteinGene-typing.html), indicating that the M protein is the most polymorphic bacterial protein. Large epidemiological studies on pharyngitis and invasive disease have been performed worldwide using the *emm* sequence typing (3, 7, 8). DNA molecular typing techniques that consider multiple genome markers have been also used for GAS genotyping, such as pulsed-field gel electrophoresis (9) and multi locus sequence typing (10). These methodologies have proved to be of particular importance to define the clonal structure of particular GAS populations (11). Recently, *emm*-cluster typing system, which groups most *emm* types into 48 different functional *emm* clusters on the basis of their structural properties, and multiple-locus variable-number of tandem repeats analysis have been proposed as promising additional GAS typing tools (12, 13). The recent advances in whole-genome sequencing (WGS) technologies with reduced costs and turnaround times, along with the development of bioinformatics’ tools able to manage the large amount of generated data, made this technology accessible to reference microbiology (14). Recently, WGS coupled with the appropriate bioinformatic pipelines proved to be a reliable tool for the assignment of *emm* types and subtypes from genomic data (15–17).

Available molecular epidemiological data have been used for the design of a GAS vaccine. Despite the lack of licensed GAS vaccines, several vaccine candidates have been considered and they can be divided into M protein-based and non-M protein-based vaccines. Among the M-protein-based vaccines under clinical trial investigation there are the multivalent 26-valent and the 30-valent formulations as well as the conserved M protein vaccines (18–20); the non-M-protein-based candidate vaccines, at various stages of development, include either cell wall or several secreted virulence factors (21, 22).

The present review aimed to provide a picture of the *emm*-type distribution among invasive GAS (iGAS) strains in high-income countries of the Western world, such as Europe and North America, retrieved from the literature since the year 2000. This study had two prominent objectives: to update the picture on the most prevalent *emm* types causing invasive infections for epidemiological purposes and, second, to provide a scenario of the possible efficacies of the under-development M-based vaccine formulations.

## Methodology

### Search Strategy and Study Selection Criteria

We searched for studies that described the epidemiology of invasive GAS based on *emm* typing by the use of a systematic approach. Searches were done in PubMed Medline for papers published from 1st January 2000 to 31st May 2017 by the combined use of the search terms “*emm*” and “invasive.” The search was planned in order to include only GAS isolates responsible for invasive infections according to the case definition (23). Exclusion criteria included studies that involved other beta-hemolytic streptococci, not population-based surveys, outbreak, and case-report studies, surveillance studies focused to specific *emm* types or limited to the analysis of antibiotic-resistant strains. Reports considering small numbers of strains (approximately not more than 40 GAS strains) were also not considered, except for those papers that were the only representative of a given European Country. Moreover, all studies that were part of major cited studies and reviews were also excluded from the analysis. Those studies for which clear data were not available, such as studies with uncertainty on the time period of the collection of strains, on the origin of strains, on the *emm*-type distribution, on the geographical origin, or studies involving strains collected across 2000 but starting earlier in 1990s and for which *emm* types after 2000 were not precisely provided, were not considered in this review. Finally, the search was restricted to only papers in English language. Overall, 264 articles were retrieved. A total of 38 articles, of which 31 and 7 from European and North American studies, respectively, were selected by the criteria described above. Five additional articles, all European studies, which were cited in the references although not retrieved by using the search criteria, were also included.

### Data Extraction and Analysis

A database was created to record the country, the period (years) of isolation, the geographic area involved (local, regional, or a nationwide survey), the group ages of patients affected by iGAS, the overall number of genotyped iGAS and the *emm*-type distribution. Only one isolate per patient was included and the relative frequency of each *emm* type was determined or extrapolated from each study. All the information obtained was incorporated into an Excel file and summarized in Table S1 in Supplementary Material for European studies and Table S2 in Supplementary Material for North American studies.

## Results

### Europe

All countries located in the European continent were considered. Therefore, besides the 28 European Union (EU) countries, Iceland, Liechtenstein, Norway, and Switzerland were included in the search.

**Figure 1:**
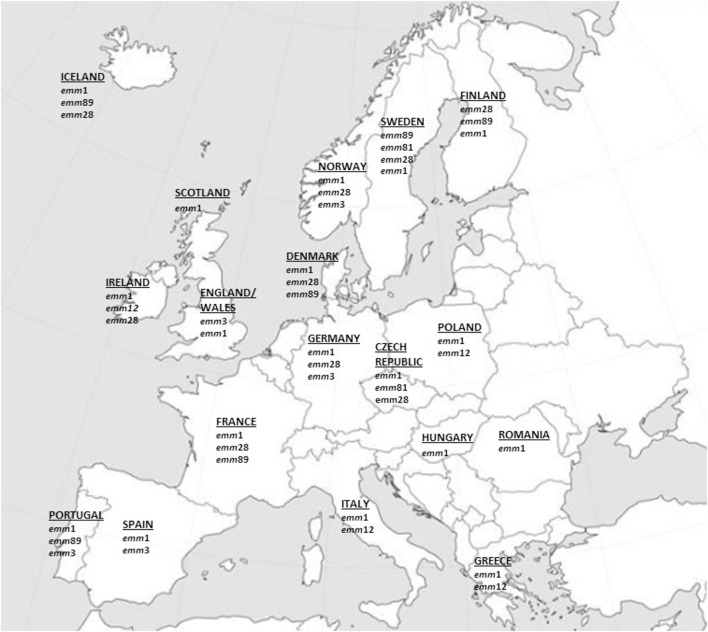
Most prevalent invasive GAS *emm* types in Europe since 2000 onward. The major *emm* types (accounting for approximately ≥10% of all *emm* types) from European epidemiologic surveys that met the criteria used in this review are represented.

No data on iGAS *emm* types were retrieved from 15 countries (Austria, Belgium, Bulgaria, Croatia, Cyprus, Estonia, Latvia, Lithuania, Luxembourg, Malta, Netherlands, Slovakia, Slovenia, Liechtenstein, and Switzerland) while for the other 17 countries at least one study on iGAS *emm*-type distribution could be retrieved and taken into consideration. The findings of the most prevalent *emm* types encountered in each European Country are summarized in the Figure 1 and in Table S1 in Supplementary Material.

#### Northern Countries

##### Denmark

Three studies were available for Denmark covering the period 2001–2011. Overall, the major *emm* types were *emm*1, 28, 89, and 3, with the latter commonly found only since the year 2003 onward. The most recent study described a nationwide laboratory-based surveillance between years 2005 and 2011 and it analyzed 910 iGAS isolates (24). A total of 58 different *emm* types were identified, with *emm* types 1 (26%), 28 (17%), 3 (13%), 89 (11%), and 12 (9%) representing 76% of all isolates. *emm*1 predominated in most years, except in the year 2010 when *emm*28 was the most prevalent, and 2011 when *emm*1 predominated along with *emm*3 (24).

Immediately before, in the period 2003–2004, another national active surveillance study on 278 iGAS infections, as a part of the Strep-EURO project, demonstrated that *emm*28 (26%) and *emm*1 (24%) accounted for approximately 50% of strains, followed by *emm*3 (11%), *emm*89 (7%), and *emm*12 (5.5%) (25). Fluctuation of the *emm*-type distribution was observed over time but *emm*28 that was constant over time, whereas *emm*1 decreased and *emm*3 increased (25).

Another report was a nationwide prospective surveillance study performed between January 2001 and August 2002 on GAS collected from patients with invasive infections, noninvasive infections, and asymptomatic carriers. This study revealed that among the 200 invasive isolates, a total of 27 different *emm* types were found, of which *emm*1, *emm*28, *emm*12, *emm*6, and *emm*4 accounted for 69% of the total *emm* types (26). *emm*1 and *emm*28 alone constituted 32 and 20% of the total *emm* types, respectively, with *emm*1 that significantly increased over 2002 (26).

##### Finland

Four studies met the criteria used in this review. These encompassed the period 2000–2013 and the major *emm* types were *emm*28, 89, and 1. The most recent paper was a nationwide study conducted during the period 2008–2013 on 1,112 iGAS (27). A total of 72 different *emm* types were identified, of which *emm*28 (26%), *emm*89 (17%), and *emm*1 (12%) were the most common types (27). An increase of erythromycin and clindamycin resistance was observed at that time that paralleled with the emergence of the novel clone *emm*33, detected for the first time in 2012 (27). This study revealed that *emm*89 increased from 2008 to 2013, *emm*1 decreased while *emm*84, that was common in 2008, was not detected in 2013 (27).

Another nationwide survey was performed to assess the population-based incidence and outcomes of pediatric iGAS infections (28). During the period of 1996–2010, a total of 151 children with iGAS infection were identified (28). Overall, 60 isolates were genotyped and *emm*1 strains were the most prevalent in the years 2000–2010, showing a typical fluctuation by herd immunity, followed by *emm*28, *emm*12, and *emm*89 (28).

In 2006, an increase of iGAS disease was observed (29). Therefore, a nationwide surveillance study was conducted in the period 2004–2007. A total of 1,318 cases of iGAS were identified displaying 46 different *emm* types. The most common *emm* types were 28 (21%), 1 (16%), 84 (10%), 75 (7%), and 89 (6%), accounting for 60% of isolates (29). Fluctuations in *emm*-type distribution were observed over the study period.

One regional study on the *emm*-type distribution among invasive beta-hemolytic streptococci, including GAS, conducted in 1-year period (2008–2009) in Pirkanmaa Health District (western Finland) revealed that among 50 iGAS blood or deep tissue isolates the most common *emm* types were *emm*77 (32%), *emm*28 (22%), *emm*89 (14%), and *emm*1 (8%) (30).

##### Iceland

Only one study was retrieved by using the chosen criteria; it comprised 288 iGAS cases from 1975 to 2012 (31). Overall, 226 GAS isolates were *emm* typed, of which 132 isolates were recovered since 2000 onward; they displayed a total of 11 different *emm* types with *emm* types 1 (26%), 89 (17%), 28 (14%), 12 (11%), 4 (8%), 3 (6%), and 22 (6%), as the more prevalent (31). *emm*1 strains most likely caused severe infections, *emm*4 was significantly more common among children, whereas *emm*28 was identified solely among adults (31).

##### Norway

Two nationwide and three regional surveillance studies on iGAS in Norway have been reported since 2000. The studies comprised iGAS collected over the period 2000–2014 and the major *emm* types identified were *emm*1, 28, 3, and 89.

The most recent national study regarded an epidemiological surveillance on iGAS infections from 2010 to 2014 (32). Overall, 756 iGAS isolates were analyzed and 52 different *emm* types were identified (32). *emm*1 (27%), followed by *emm*89 (12%), *emm*12 (9%), *emm*3 (8%), and *emm*28 (8%) were the most prevalent types and accounted for 64% of all isolates (32).

The second nationwide study was conducted on 262 invasive isolates collected during the years 2006 and 2007. Overall, 29 different *emm* types, with 37 *emm* subtypes, were identified (33). The most prevalent *emm* types were *emm*28 (19.5%), *emm*1 (14.1%), *emm*82 (14.1%), *emm*12 (11.8%), *emm*4 (8.0%), *emm*3 (6.1%), and *emm*87 (5.7%). A significant increase in the prevalence of *emm*6, overrepresented in males, was noted (33). Comparing the *emm*-type distribution between 2007 and 2014, *emm*28 dominated in 2007 then decreased, while *emm*1 increased over time (32, 33). Five out of the six most frequent *emm* types in 2007 remained among the top six *emm* types from 2010 to 2014, but *emm*82, the third most frequent *emm* type in 2007 was completely absent in the years 2010–2014, replaced by *emm*89 which increased (32, 33).

The three regional epidemiological studies on iGAS were conducted in Western Norway. The largest regional study was focused on all beta-hemolytic groups (A, C, and G streptococci) isolated over a 15-years period (1999–2013) (34). Among the 209 iGAS, 26 different *emm* types and 34 *emm* subtypes were identified (34). The most frequent *emm* types were consistent along the entire period of study and they were *emm*1 (23.9%), *emm*28 (14.3%), *emm*3 (13.9%), *emm*89 (6.2%), *emm*11 (5.7%, responsible for an outbreak), and *emm*4 (5.2%) (34). The other two studies considered a lower number of strains and involved beta-hemolytic group A, C, and G streptococci. One study was performed on necrotizing soft tissue infections caused by beta-hemolytic group A, C, and G streptococci during the period 2000–2009 (35). Overall, 42 iGAS isolates were analyzed and the most prevalent *emm* types were, in decreasing order, *emm*1 (31%), *emm*3 (11.9%), and *emm*4 (9.5%) (35). The other study was on invasive A, C, and G streptococcal infections observed in the years 2006–2009 and involved a total of 59 iGAS (36). The six most common *emm* types accounted for 81% of the isolates and they were *emm*28 (22%), *emm*3 (17%), *emm*1 (15%), *emm*12 (10%), *emm*82 (10%), and *emm*89 (7%), with the first three *emm* types responsible for more than 50% of the infections, associated with severe outcome (36).

##### Sweden

Only one study met the selected criteria; it was an enhanced surveillance study on iGAS (746 isolates) performed in the years 2002–2004, of which the majority (94%) were isolated from bacteremia (37). The most abundant *emm* types were *emm*89 (15.7%) and *emm*81 (14.5%), followed by *emm*28 (13.9%), *emm*1 (11.9%), *emm*12 (6.3%), *emm*77 (5.9%), and *emm*4 (5.9%).

##### England and Wales

Two studies from United Kingdom were included in this survey, of which one on a large collection of iGAS isolated in 2014, the other on a small collection of strains recovered in the early 2000s. The bigger study was conducted after an increase of scarlet fever episodes observed in 2014 in England and involved 252 iGAS. *emm*3 was the most prevalent *emm* type representing 28.2% of all iGAS, followed by *emm*1 (22.6%), *emm*89 (9.1%), *emm*12, and *emm*28 (7.1% each) (38).

The other report was a small surveillance study performed on blood GAS isolates obtained from injecting drug users compared to isolates from non-drug users at the Royal Sussex Hospital in Brighton over the period 2000–2003 (39). Overall, 44 iGAS were available for the study and the most frequent *emm* types were *emm*83 (25%), *emm*82 (18.2%), *emm*1 (15.9%), *emm*89 (13.6%), and *emm*87 (11.4%) (39). Differences in *emm*-types distribution were noted between injecting drug users and non-drug users, with *emm*82 and *emm*83 almost exclusively found in the injecting drug users population (39).

##### Scotland

Only one national study from Scotland was retrieved; it reported the distribution of GAS *emm* types from invasive and noninvasive sites from all age groups patients over a 4-years period (2011–2015). This study revealed that among 329 iGAS strains recovered from sterile sites *emm*1 (66% of all iGAS) was the most prevalent and strongly associated with iGAS infections (40). The other most common *emm* types identified in invasive infections were, in decreasing order, *emm*76 (7.4%), *emm*89 (6.7%), and *emm*3 (5.8%).

##### Ireland

The only study from Ireland that met the chosen criteria documented an increased incidence of iGAS over in the years 2012–2013; *emm*1 dominated over the entire study period (41). In 2012, 176 iGAS were genotyped and the most predominant types were *emm*1 (48.6%), *emm*12 (9.2%), and *emm*28 (7.3%). In 2013, a further increment in iGAS infections was noted and it was associated with a notable increase in *emm*3 isolates in the first half of 2013, reaching the rate of 22% from that of 4% observed in 2012 (41). The relative percentages of *emm* types found over the entire study period were not clearly indicated.

#### Eastern Countries

##### Czech Republic

The only study on iGAS was an epidemiological survey on 215 iGAS obtained from 34 hospitals throughout the Country, isolated during the years 2001–2005 (42). The emergence of the uncommon GAS *emm*53 type was reported, with the highest proportion observed during 2003, possessing resistance to macrolides (42). The most prevalent *emm* type was *emm*1, followed by *emm*81, *emm*28, *emm*53, *emm*3, and *emm*66, although the relative frequency of each *emm* type was not reported (42).

##### Hungary, Poland, and Romania

One national surveillance study from each Country has been retrieved according to the used criteria, all three including only a limited number of iGAS isolates.

The nationwide laboratory-based surveillance study from Hungary regarded the molecular characterization of 26 iGAS isolates obtained in the years 2004 and 2005. The most prevalent *emm* types encountered in this study were *emm*1 (50%) and *emm*80 (19.2%) (43).

The study from Poland was a national laboratory-based survey involving 17 different hospitals distributed in different part of the Country (44). The major limitation of this study for our analysis was the lack of data on the *emm*-type distribution obtained since 2000 onward, but considering that it was the only molecular study on iGAS from this country, it was included. Overall, 41 iGAS clinical isolates were obtained between the years 1997 and 2005, with a total of 23 different *emm* types identified, of which *emm*1 and *emm*12 (19.5% each) were the most frequent, followed by *emm* 81 (7.3%) (44).

A total of 33 iGAS obtained from 8 different Romanian districts during the years 2003–2004 were genotyped as a part of the Strep-Euro project; the predominant *emm* types were *emm*1 (15.1%), *emm*76, and *emm*81 (12.1% each) (45).

#### Western Countries

##### France

Several studies on the epidemiology and *emm*-type distribution of iGAS isolates recovered from 2000 to 2013 met the criteria used in this review. Overall, the most prevalent *emm* types were *emm*1, 28, and 89.

The first report was a molecular epidemiology study on iGAS isolated from French children between the years 1999 and 2006 (46). Among the 74 strains available for the study, 31 different *emm* types were identified, associated with specific virulence genes. *emm* types 1 (25.7%), 89 (9.5%), 3 and 4 (8.1% each), and 6, 12, and 28 (6.7% each) predominated (46).

Another report investigated the characteristics of GAS responsible for meningitis in adults from 2003 to 2013 (47). Overall, 63 GAS isolates were identified and four predominant *emm* types were found: *emm*1 (44%), *emm*28 (12.7%), and *emm*3 and *emm*6 (11.1% each) (47).

Four other studies on French iGAS strains isolated in the years 2006–2011 found *emm*1 as the predominant type. Two of these studies were nationwide surveillances on a large number of strains recovered from all age group patients in France (48, 49); in particular, one report assessed the epidemiology of 623 iGAS strains isolated in 2007, the other study involved more than 2,600 iGAS collected in the period 2007–2011. The most prevalent *emm* types were quite the same between studies, with slight differences: *emm*1 predominated, followed by *emm*28 or *emm*89, *emm*4, and *emm*12 (48, 49). The other two French studies were a report on 1,542 iGAS recovered from adults in the years 2006 and 2010 (50) and 125 iGAS strains isolated from children in the period 2009–2011 (51). The first study identified *emm*1 as the most common type accounting for 24% of the isolates, followed by *emm*28 (17%), *emm*89 (15%), *emm*4, *emm*3, and *emm*12 (5% each); the other study revealed that *emm*1 predominated (24.8%), followed by *emm*12 and *emm*28 (15.2% each), *emm*6 (12%), and *emm*3 (9.6%).

##### Germany

Two German studies have been included in this review, covering the years 2000–2009. *emm* types 1, 28, and 3 predominated. One major study was a nationwide laboratory-based surveillance to analyze the epidemiology of varicella-associated iGAS infections over the years 1996–2009 (52). Overall, 1,342 iGAS were analyzed and the most abundant *emm* type was *emm*1 (32.6%), followed by *emm*28 (13.8%), *emm*3 (8.3%), *emm*12 (6.1%), and *emm*89 (5.5%). *emm*1, *emm*12, and *emm*4 types, possessing a high rate of the virulence gene *ssa*, were significantly related to varicella-positive isolates (52).

The second nationwide laboratory-based surveillance study of iGAS was performed on a total of 586 strains, mostly recovered from blood and skin infections, during the years 2003–2007 (53). Also in this study, *emm*1 strongly dominated (30.5%) followed by *emm*28 (18.3%), *emm*3 (9.6%), and *emm*12 and *emm*89 (7% each) (53).

#### Southern Countries

##### Greece

Two national surveillance reports have been selected, covering the period 2003–2007, and they both indicated *emm*1 and *emm*12 as the most prevalent types. A multicenter laboratory-based surveillance study was conducted between the years 2003 and 2007; among the 138 iGAS available for genotyping the two most prevalent *emm* types were *emm*1 (28.2%), mainly isolated in adults, and *emm*12 (8.5%) (54). The other study was a multicenter surveillance involving a total of 101 isolates obtained between the years 2003 and 2005 and the most common *emm* types were *emm*1 (26.5%), *emm*12 (8.9%), *emm*4, *emm*6, and *emm*95 (5% each) (55).

##### Italy

Only one study from Italy was retrieved on iGAS isolates from 2000 onward, a 3-year (between 2003 and 2005) nationwide enhanced surveillance study as part of the StrepEuro surveillance; it comprised 89 iGAS, where *emm*1 largely predominated (19%), followed by *emm*12 (12%), *emm*3 (10%), *emm*4 (9%), *emm*18 (8%), and *emm*6 (5%) (56).

##### Portugal

Two major consecutive surveillance studies involving iGAS have been performed in the period 2000–2009. *emm*1, *emm*3, *emm*6, and *emm*89 represented the four most common types, with differences in their order of frequency.

The most recent study was conducted on iGAS isolated between 2006 and 2009 (57). A total of 191 iGAS were characterized with *emm*1 (29.3%), *emm*89 (12.6%), *emm*3 (10.5%), *emm*6 (7.8%), accounting for 60% of the isolates (57). In this study, it was observed an intra-clonal diversity of the superantigen genes profiles, with evidence of specific superantigen gene loss and acquisition (57).

The second study was a multicenter laboratory-based surveillance of iGAS conducted over a period immediately before the aforementioned survey, between the years 2000 and 2005 (11). Among 160 non-duplicate iGAS isolates, the six most common *emm* types were *emm*1 (20%), *emm*3 (9.3%), *emm*89 (8.1%), *emm*6, *emm*28, and *emm*64 (6.9% each) (11).

##### Spain

Only one study met the criteria and it was a regional survey on invasive strains recovered from two regions in Spain between the years 1998 and 2009. Among the 215 isolates, *emm*1, associated with *speA* and *ssa* genes, largely predominated (27.9%) and was responsible for the majority of fatal outcomes, followed by *emm*3 (9.8%), *emm*4 (6.5%), and *emm*28, *emm*12, and *emm*89 (6% each) (58).

### North America

**Figure 2:**
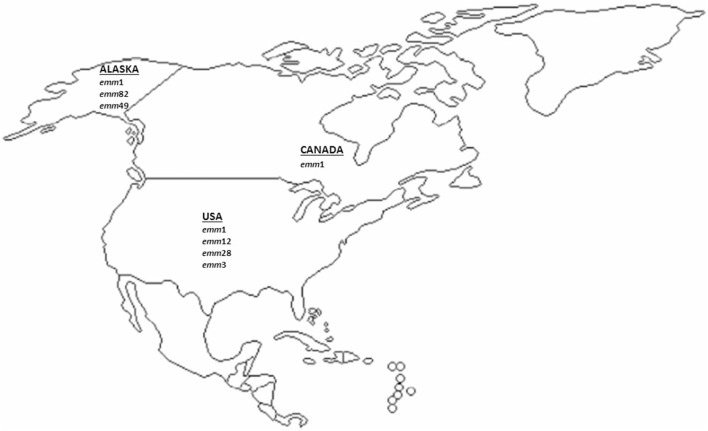
Most prevalent invasive GAS *emm*-types described in North America (US and Canada) since 2000 onward. The major *emm* types (accounting for approximately ≥10% of all *emm* types) from the US and Canadian studies included in this analysis are represented.

Four and three studies on *emm*-type distribution of iGAS from US including Alaska and from Canada, respectively, were considered in this review. The results of the *emm*-type distribution and their relative frequencies reported in North America are depicted in Figure 2 and in Table S2 in Supplementary Material.

#### United States

Three major studies on iGAS collected by the Centers for Disease Control and Prevention’s Active Bacterial Core surveillance, a population-based network, including geographically different states from US, were retrieved. Altogether, these three studies encompassed a time period of 13 years, from 2000 to 2012. *emm*1 was the predominant type in all studies and four other *emm* types, namely *emm*3, *emm*12, *emm*28, and *emm*89, were among the most common types, with some fluctuations over time.

The most recent survey was conducted on 8,200 iGAS obtained over the period 2005–2012 in which *emm*1 was the most prevalent type (22%), followed by *emm*12 (9%), *emm*28 (8%), *emm*89, and *emm*3 (7% each) (59). *emm* types included in the currently proposed 30-valent vaccine accounted for 91% of the cases (59).

Another study was performed on 4,350 iGAS recovered in 10 different US sites during the period 2000–2004 (8). *emm*1 largely dominated at a rate of 18.2%, followed by *emm*3, *emm*28, *emm*12 (9% each), and *emm*89 (6%), all together accounting for 55% of the isolates (8). In this study, the *emm* types included in the proposed 26-valent vaccine accounted for 79% of all cases (8).

The first population-based study conducted on iGAS recovered since 2000 onward involved nine different States and 1,061 invasive strains recovered during the years 2000 and 2001 (60). The six most prevalent *emm* types were *emm*1 (18.2%), *emm*3 (10.2%), *emm*12 (8.5%), *emm*28 (7.9%), *emm*82 (5.9%), and *emm*89 (5.5%), which accounted for approximately 55% of the isolates (60).

In Alaska, the Arctic Investigations Program set up a population-based laboratory surveillance program on iGAS infections during the period 2001–2013 (61). Overall, 516 cases of iGAS infections were reported, and 422 isolates were available for microbiological analysis and typing. A total of 51 different *emm* types were identified, of which *emm*1 was the most common (11.1%) followed by *emm*82 (8.8%), *emm*49 (7.8%), *emm*12, and *emm*3 (6.6% each), *emm*89 (6.2%), and *emm*108 (5.5%) (61). The *emm* types included in the 30-valent M protein vaccine accounted for 78% of isolates (61).

#### Canada

Overall, three studies on molecular epidemiology of iGAS infections were found, encompassing a 9-years period (between 2006 and 2014), with large geographical differences in the *emm*-type distribution.

A recent epidemiological study on iGAS strains from remote indigenous communities in Northwestern Ontario, over the period 2009–2014, indicated that among 46 invasive isolates, 14 different *emm* types were identified, with *emm*-type variability observed over time. The most common *emm* types were *emm*114 (17.4%), *emm*11 (15.2%), *emm*118 (13.0%), *emm*68, and *emm*82 (10.9% each) (62).

Another regional surveillance study of iGAS in the province of Ontario started because of an outbreak of iGAS infections due to *emm*59 strains occurring in Thunder Bay District, North-Western Ontario in 2008. All iGAS obtained in Thunder Bay District from 2011 to 2013 were studied and they were compared to iGAS strains recovered during the same period from the metropolitan area of Toronto/Peel and the province of Ontario (63). Most iGAS cases isolated in the Thunder Bay District were caused by strains belonging to skin or generalist *emm* types, while those from the province of Ontario and the Toronto metropolitan area were caused by *emm* types frequently associated with invasive GAS infections (63). In particular, among the 66 iGAS obtained from Thunder Bay District the six most prevalent *emm* types were *emm*87 (12.3%), *emm*82 (10.8), *emm*1, *emm*101, and *emm*83 (9.2% each), and *emm*114 (7.7%). By contrast, from the metropolitan area of Toronto/Peel and the rest of province of Ontario *emm*1 predominated (23.5%), followed by *emm*89 (12.7%), *emm*3 (11.3%), *emm*12 (8.2%), and *emm*28 (5.7%) (63).

The third study was a nationwide surveillance conducted from January 2006 to December 2009 and included 4143 iGAS obtained from 10 provinces and 3 territories in Canada (64). Among all iGAS cases, 539 (13%) were attributed to *emm*59, mainly circulating in the province of Ontario. *emm*1, *emm*28, *emm*3, *emm*89, and *emm*12 were other well-represented types, although the relative percentages of each *emm* type were not provided (64).

## Discussion and Conclusion

In this review, we provided a picture of the most prevalent *emm* types among iGAS reported in Europe and North America from the year 2000 to May 2017 (last updated on June 1st 2017). It is interesting to note that the major *emm* types were almost the same in all European countries and in US. *emm*1 largely represented the dominant type in all countries, and in only few cases it was replaced by other common *emm* types, such as *emm*28, reported in Denmark, Finland, and Norway, *emm*89 and *emm*77 reported in Sweden and Finland, respectively, and *emm*3 in the United Kingdom. Other common *emm* types were *emm*28, *emm*89, *emm*3, *emm*12, *emm*4, and *emm*6, with differences in their prevalence among countries. Some “uncommon” *emm* types were sporadically encountered as prevalent types, as for *emm*84 and *emm*119 in Finland; *emm*11 in Norway; *emm*75 in Finland, Norway, and Spain; *emm*81 in Finland, Sweden, Czech Republic, Hungary, Poland, and Romania; *emm*82 in Norway and Alaska; *emm*66 in Norway and Czech Republic; *emm*53 in Finland and Czech Republic; *emm*18 in Italy; *emm*64 in Portugal; *emm*49 and *emm*108 in Alaska; and *emm*59 in Canada. Interestingly, *emm*81 was commonly found in all Eastern European countries, suggesting the likely spread of this type between neighboring countries.

Overall, the major *emm* types (*emm*1, *emm*28, *emm*89, *emm*3, *emm*12, and *emm*4, and *emm*6) accounted to only up 70% of the total isolates. Therefore, it is of some concern that several other different *emm* types that are less represented nevertheless accounted for the remaining 30–50% of the cases. Some of these minor *emm* types could emerge and become among the most prevalent ones in the future.

In North America, *emm*1 was the most prevalent type in most national studies from US, followed by *emm*12, *emm*28, and *emm*3 but in regional remote communities in Canada other uncommon *emm* types predominated, such as *emm*11, *emm*87, *emm*101, *emm*114, and *emm*118.

It is important to be aware of the increase in iGAS infections with associated mortality observed in the last years in several European countries (40, 65–67), reinforcing the need of an European multinational surveillance network that could compensate the scattered available information on the iGAS disease burden (68).

Data on the *emm*-type distribution of population-based GAS surveillance have been also used for the development of M-multivalent GAS vaccine candidates. The 26-M-valent and 30-M-valent vaccines have been developed in order to maximize the “coverage” of circulating *emm* types (19). Epidemiologic surveys suggest that the 26-valent vaccine would provide good coverage of circulating GAS strains in industrialized countries (over 72%) but poor coverage in many developing countries due to differences in *emm* distribution (3). Similarly, the 30-valent vaccine has a limited coverage in many developing countries where GAS infections are endemic but this inconvenience is likely mitigated by the demonstration that the 30-valent vaccine induces protection not only against the *emm* types contained in the vaccine but it also cross-reacts to some non-vaccine *emm* types (69).

It is of some concern that some *emm* types found to be well-represented in some European studies are not included into the vaccine formulations, as is the case of the *emm* types 53, 64, 66, 84, 108, and 119; on the other end, sporadic *emm* types as *emm* 49, 81, and 82 that were identified in the European studies or *emm* types 87, 101, and 118 isolated in regional Canadian surveys are included in one or the other of the two vaccines (19, 70).

Fluctuations in *emm*-type distribution have been attributed to multiple factors, such as different frequencies of the most prevalent circulating clones, influence of the herd immunity, different clinical manifestations of iGAS infections associated with specific *emm* types, age, racial, and seasonal differences (25, 28, 32). Recently, seasonal, geographic, and temporal trends of specific *emm* clusters associated with iGAS infections have been found, due to a probable different capacity for transmission or infection (71).

The factors contributing to the fluctuations and/or success of specific epidemic clones in invasive diseases have always gained a priority interest in term of prevention control and the study of the dynamics of GAS population. The predominance of particular *emm* types in invasive disease could likely be a consequence of the high prevalence in the entire population, as demonstrated for *emm*1, suggestive of the better success of specific clones as well-adapted human colonizers (72). In the last few years, the use of WGS has proved to be a useful way to investigate the evolutionary history of these highly successful GAS epidemic or pandemic clones belonging to specific *emm* types. Specific virulence factors, such as streptococcal pyrogenic exotoxin (spe) A and its alleles, streptokinase, streptolysin O (slo), NAD glycohydrolase (nga), have been associated with shaping and spreading of successful strains. For example, the emergence of the *emm*1 epidemic clone has been associated with three consecutive gene transfer events, which is the acquisition of Dnase sdaD2, expression of speA2 and upregulation of the virulence factors slo and nga (73). Similarly, for *emm*89, the emergence of a third clonage lineage (clade 3 clone) by modification in the nga/slo locus and loss of the capacity to synthesize the capsule is the cause of an ongoing epidemic of invasive infections in Europe and North America (74). Another recent study from England reported an increase in iGAS infections associated with *emm*3 isolates. By analysis of the whole-genome single-nucleotide polymorphism-based phylogeny the main factor responsible for this upsurge seemed to be associated with the expansion of a genetic lineage characterized by the presence of a prophage carrying speC exotoxin and spd1 DNAase genes and the loss of two other prophages considered typical of the *emm*3 strains (75).

Notwithstanding the importance of loss or acquisition of genes by horizontal transfer (mostly mediated by bacteriophage or pathogenicity islands) as a fundamental evolutionary forces shaping the structure of GAS genomes, it is also evident that there is not a strict correlation between defined patterns of exogenous elements-associated virulence genes and invasiveness or disease severity. The main contribution to the generation and expansion of *emm*-type specific invasive clones seems to be more related to mutations in some important genetic regulatory circuits governing the global expression of GAS virulence. Among them, there are the two components system *CovR/S* (76), the regulator of protease B *ropB* (77), the regulator of Cov *RocA* (78), the *Fas* system (79), and the *RofA*-like protein IV regulator *RivR* (80).

In this context, it is clear how *emm* type (i.e., M serotype) continues to be the GAS epidemiological reference marker for tracking the clonal radiation of epidemic lineage from a common genetic background. The recent advances of WGS technologies with reduction in terms of costs and reduced turnaround times have been proposed as the method of choice to type strains, GAS included. The design of bioinformatics components to emulate current methods, however, is still laboratory-restricted and standardization of different steps still needs improvements to ensure backward compatibility with the classical “gold standard” typing methods and schemes (15, 17). The potential of WGS data analysis is huge as already demonstrated also for GAS where temporal and geographic relatedness between GAS isolates could be deduced (16). These new “omics” approaches may also provide rapid assessment of outbreaks by discriminating between closely related isolates, and produce extensive data on the virulome, including the associated regulatory mechanisms, and the antibiotic resistome (81, 82). Hopefully, this review can be a reference for all those who work in the field of molecular epidemiology of GAS and may represent a milestone also to those who are approaching the problem using the recent next generation sequencing methodologies.

In any case, continuous surveillance of the *emm*-types distribution of iGAS is strongly encouraged to monitor the prevalent *emm* types responsible for the disease burden and the potential coverage of the under-development multivalent vaccines.

## Author Contributions

GG and RC conceived the project. GG and RC searched the database for potentially eligible articles, extracted the data, and performed the analyses. GG, LV, and RC interpreted the results and wrote the manuscript. All the authors reviewed the final version of the manuscript prior to submission for publication.

## Conflict of Interest Statement

The authors declare that the research was conducted in the absence of any commercial or financial relationships that could be construed as a potential conflict of interest.
