# Supplementary material for: Prevalent emm Types among Invasive GAS in Europe and North America since Year 2000
Source: Front Public Health. 2018 Mar 9;6:59. doi: 10.3389/fpubh.2018.00059 (PMC5890186; doi:10.3389/fpubh.2018.00059)
Supplement: Supplementary file 1 [file table_1.PDF]

Table S1. Most prevalent *emm* types (> 5%) among invasive GAS isolates reported in Europe, Country-by-Country, since 2000 (data reported from the literature, last access May 2017).

Country	Period	Study design	Age group	No. invasive isolates/cases	emm types (%)	Reference
**Northern Countries**						
**Denmark**	2005-2011	National	All age groups	910	*emm*1 (26%)<br>*emm*28 (17%)<br>*emm*3 (13%)<br>*emm*89 (11%)<br>*emm*12 (9%)	Lambertsen et al, Clin Microbiol Infect, 2013
	2003-2004	National	All age group	278	*emm*28 (26%)<br>*emm*1 (24%)<br>*emm*3 (11%)<br>*emm*89 (7%)<br>*emm*12 (5.5%)	Luca-Harari et al, J Clin Microbiol, 2008
	2001-2002	National	All age group	200	*emm*1 (32.5%)<br>*emm*28 (20%)<br>*emm*89 (10%)<br>*emm*12 (5.5%)<br>*emm*4 (5%)	Ekelund et al, J Clin Microbiol, 2005
**Finland**	2008-2013	National	All age group	1122	*emm*28 (26.5%)<br>*emm*89 (17.2%)<br>*emm*1 (11.8%)<br>*emm*12 (6.9%)<br>*emm*119.1 (6.1%)	Smit et al, Eur J Clin Microbiol Infect Dis, 2015
	2000-2010	National	Children	52	*emm*1 (38.5%)<br>*emm*28 (17.3%)<br>*emm*12 (13.5%)<br>*emm*89 (9.6%)	Tapiainen et al, Pediatr Infect Dis J, 2016
	2008-2009	Regional	All age group	50	*emm*77 (32%)<br>*emm*28 (22%)	Vähäkuopus et al, Eur J Clin Microbiol Infect Dis, 2012

					*emm*89 (14%)<br>*emm*1 (8%)	
	2004-2007	National	All age group	602	*emm*28 (21%)<br>*emm*1 (16%)<br>*emm*84 (10%)<br>*emm*75 (7%)<br>*emm*89 (6%)	Siljander et al, Eur J Clin Microbiol Infect Dis, 2010
**Iceland**	2000-2012	National	All age groups	132	*emm*1 (26%)<br>*emm*89 (17%)<br>*emm*28 (14%)<br>*emm*12 (9.3%)<br>*emm*4 (11%)<br>*emm*3 (6%)<br>*emm*22 (6%)	Olafsdottir et al, Euro Surveill, 2014
**Norway**	2010-2014	National	All age group	756	*emm*1 (27%)<br>*emm*89 (12%)<br>*emm*12 (9%)<br>*emm*3 (8%)<br>*emm*28 (8%)<br>*emm*4 (6.9%)<br>*emm*66 (5.7%)	Naseer et al, Eur J Clin Microbiol Infect Dis, 2016
	2006-2007	National	All age group	262	*emm*28 (19.5%)<br>*emm*1 (14.1%)<br>*emm*82 (14.1%)<br>*emm*12 (11.8%)<br>*emm*4 (8%)<br>*emm*3 (6.1%)<br>*emm*87 (5.7%)	Meisal et al, J Clin Microbiol, 2010
	1999-2013	regional	All age group	209	*emm*1 (23.9%)<br>*emm*28 (14.3%)<br>*emm*3 (13.9%)<br>*emm*89 (6.2%)<br>*emm*11 (5.7%)<br>*emm*4 (5.2%)	Oppegaard et al, Clin Microbiol Infect, 2014

	2006-2009	regional	Mainly adults	59	*emm*28 (22%)<br>*emm*3 (16.9%)<br>*emm*1 (15.2%)<br>*emm*12 (10.2%)<br>*emm*82 (10.2%)<br>*emm*89 (6.8%)<br>*emm*75 (5.1%)	Kittang et al, Clin Microbiol Infect, 2009
	2000-2009	regional	Adults	42	*emm*1 (31%)<br>*emm*3 (11.9%)<br>*emm*4 (9.5%)	Bruun et al, Clin Microbiol Infect, 2013
**Sweden**	2002-2004	National	All age group	746	*emm*89 (15.7%)<br>*emm* 81 (14.5%)<br>*emm* 28 (13.9%)<br>*emm* 1 (11.9%)<br>*emm* 12 (6.3%)<br>*emm* 77 (5.9%)<br>*emm* 4 (5.9%)	Darenberg et al, Clin Infect Dis, 2007
**England and Wales**	2014	national	not available	252	*emm*3 (28.2%)<br>*emm*1 (26.6%)<br>*emm*89 (9.1%)<br>*emm*12 (7.1%)<br>*emm*28 (7.1%)	Chalker et al, BMC Genomics, 2017
	2000-2003	Local	Adults	44	*emm*83 (25%)<br>*emm*82 (18.2%)<br>*emm*1 (15.9%)<br>*emm*89 (13.6%)<br>*emm*87 (11.4%)<br>*emm*12 (6.8%)<br>*emm*81 (6.8%)	Curtis et al, J Infect, 2007
**Scotland**	2011-2015	National	All age group	329	*emm*1 (66%)<br>*emm*76 (7.4%)<br>*emm*89 (6.7%)<br>*emm*3 (5.8%)	Lindsay et al, J Med Microbiol, 2016

**Ireland**	2012-2013	National	All age group	176	*emm*1 (n.a.)[a]<br>*emm*12 (n.a.)[a]<br>*emm*28 (n.a.)[a]<br>*emm*3 (n.a.)[a]	Meehan et al, Euro Surveill, 2013
**Eastern Countries**						
**Czech Republic**	2001-2005	National	All age group	215	*emm*1 (n.a.)[a]<br>*emm*81 (n.a.)[a]<br>*emm*28 (n.a.)[a]<br>*emm*53 (n.a.)[a]<br>*emm*3 (n.a.)[a]<br>*emm*66 (n.a.)[a]	Strakova et al, Clin Microbiol Infect, 2007
**Hungary**	2004-2005	National	Mainly adults	26	*emm*1 (50%)<br>*emm*80 (19.2%)<br>*emm*81 (7.7%)<br>*emm*84 (7.7%)	Krusco et al, Eur J Clin Microbiol Infect Dis, 2007
**Poland**	1997-2005	National	All age group	41	*emm*1 (19.5%)<br>*emm*12 (19.5%)<br>*emm*81 (7.3%)	Szczypa et al, J Clin Microbiol, 2006
**Romania**	2003-2004	National	All age group	33	*emm*1 (15.1%)<br>*emm*76 (12.1%)<br>*emm*81 (12.1%)<br>*emm*49 (9.1%)<br>*emm*75 (6.1%)<br>*emm*95 (6.1%)	Luca-Harari et al, J Med Microbiol, 2008
**Weastern Countries**						
**France**	2003-2013	National	Adults	63	*emm*1 (44.4%)<br>*emm*28 (12.7%)<br>*emm*3 (11.1%)<br>*emm*6 (11.1%)	Plainvert et al, Diagn Microbiol Infect Dis, 2016

	2009-2011	National	Children	125	*emm*1 (24.8%)<br>*emm*12 (15.2%)<br>*emm*28 (15.2%)<br>*emm*6 (12%)<br>*emm*3 (9.6%)	d'Humieres et al, Pediatr Infect Dis J, 2015
	2007-2011	National	All age group	2603	*emm*1 (27%)<br>*emm*28 (16%)<br>*emm*89 (13%)<br>*emm*4 (6%)<br>*emm*12 (6%)<br>*emm*3 (5%)	Plainvert et al, Arch Pediatr, 2014
	2006-2010	National	Adults	1542	*emm*1 (24%)<br>*emm*28 (17%)<br>*emm*89 (15%)<br>*emm*4 (5%)<br>*emm*3 (5%)<br>*emm*12 (5%)	Plainvert et al, Clin Microbiol infect, 2012
	2007	National	All age group	623	*emm*1 (33%)<br>*emm*89 (16%)<br>*emm*28 (10%)<br>*emm*4 (5%)<br>*emm*12 (5%)	Lepoutre et al, J Clin Microbiol, 2011
	1999-2006	National	Children	74	*emm*1 (25.7%)<br>*emm*89 (9.5%)<br>*emm*3 (8.1%)<br>*emm*4 (8.1%)<br>*emm*6 (6.7%)<br>*emm*12 (6.7%)<br>*emm*28 (6.7%)	Bidet et al, J Clin Microbiol, 2007
**Germany**	1996-2009	National	All age group	1342	*emm*1 (32.6%)<br>*emm*28 (13.8%)<br>*emm*3 (8.3%)<br>*emm*12 (6.1%)<br>*emm*89 (5.5%)	Imöhl et al, FEMS Immunol Med Microbiol, 2011

	2003-2007	National	All age group	586	*emm*1 (30.5%)<br>*emm*28 (18.3%)<br>*emm*3 (9.6%)<br>*emm*12 (7%)<br>*emm*89 (7%)	Imöhl et al, FEMS Immunol Med Microbiol, 2010
**Southern Countries**						
**Greece**	2003-2007		All age group	138	*emm*1 (28.2%)<br>*emm*12 (8.5%)	Zachariadou et al, Epidemiol Infect, 2014
	2003-2005	National	All age group	102	*emm*1 (26.5%)<br>*emm*12 (8.9%)<br>*emm*4 (5%)<br>*emm*6 (5%)<br>*emm*95 (5%)	Stathi et al, Clin Microbiol Infect, 2008
**Italy**	2003-2005	National	All age group	89	*emm*1 (19%)<br>*emm*12 (12%)<br>*emm*3 (10%)<br>*emm*4 (9%)<br>*emm*18 (8%)<br>*emm* 6 (5%)	Creti et al, J Clin Microbiol, 2007
**Portugal**	2006-2009	National	All age group	191	*emm*1 (29.3%)<br>*emm*89 (12.6%)<br>*emm*3 (10.5%)<br>*emm*6 (7.8%)<br>*emm*4 (7.2%)	Friães et al, Int J Med Microbiol, 2013
	2000-2005	National	All age group	160	*emm*1 (20%)<br>*emm*3 (9.3%)<br>*emm*89 (8.1%)<br>*emm*6 (6.9%)<br>*emm*28 (6.9%)<br>*emm*64 (6.9%)<br>*emm*12 (6.2%)	Friães et al, J Clin Microbiol, 2007

					*emm*4 (5.6%)	
**Spain**	1998-2009	Regional	All age group	215	*emm*1 (27.9%)<br>*emm*3 (9.8%)<br>*emm*4 (6.5%)<br>*emm*28 (6%)<br>*emm*12 (6%)<br>*emm*89 (6%)<br>*emm*6 (5%)	Montes et al, Eur J Clin Microbiol Infect Dis, 2011

[a]n.a., not available, the order of frequency of the major *emm* types was indicated, although their relative percentages were not clearly indicated.
