# Supplementary material for: Prevalent emm Types among Invasive GAS in Europe and North America since Year 2000
Source: Front Public Health. 2018 Mar 9;6:59. doi: 10.3389/fpubh.2018.00059 (PMC5890186; doi:10.3389/fpubh.2018.00059)
Supplement: Supplementary file 2 [file table_2.PDF]

Table S2. Most prevalent *emm* types (≥ 5%) among invasive GAS isolates reported in North America (United States, Alaska, and Canada), since 2000 (data reported from the literature, last access May 2017).

Country	Period	Study design	Age group	No. isolates/cases	emm types (%)	Reference
**United States**	2005-2012	National	All age group	8200	*emm*1 (22%)<br>*emm*12 (9%)<br>*emm*28 (8%)<br>*emm*89 (7%)<br>*emm*3 (7%)	Nelson et al, Clin Infect Dis, 2016
	2000-2004	National	All age group	4350	*emm*1 (22%)<br>*emm*3 (9%)<br>*emm*28 (9%)<br>*emm*12 (9%)<br>*emm*89 (6%)	O'Loughlin et al, Clin Infect Dis, 2007
	2000-2001	National	All age group	1061	*emm*1 (18.2%)<br>*emm*3 (10.2%)<br>*emm*12 (8.5%)<br>*emm*28 (7.9%)<br>*emm*82 (5.9%)<br>*emm*89 (5.5%)	Li et al, J Infect Dis, 2003
**Alaska**	2001-2013	Regional	All age group	422	*emm*1 (11.1%)<br>*emm*82 (8.8%)<br>*emm*49 (7.8%)<br>*emm*12 (6.6%)<br>*emm*3 (6.6%)<br>*emm*89 (6.2%)<br>emm108 (5.5%)	Rudolph et al, J Clin Microbiol, 2016
**Canada**	2009-2014	Regional, Northwestern Ontario	All age group	46	*emm*114 (17.4%)<br>*emm*11 (15.2%)<br>*emm*118 (13%)<br>*emm*68 (10.9%)<br>*emm*82 (10-9%)<br>*emm*1 (6.5%)	Bocking et al, Open Forum Infect Dis, 2016

				*emm*101 (6.5%)	
2011-2013	Regional- Thunder Bay District	All age group	117	*emm*87 (12.3%)<br>*emm*82 (10.8%)<br>*emm*1 (9.2%)<br>*emm*101 (9.2%)<br>*emm*83 (9.2%)<br>*emm*114 (7.7%)	Athey et al, J Clin Microbiol, 2016[a]
2011-2013	Regional – Toronto metropolitan area and rest of Ontario	All age group	756	*emm*1 (23.5%)<br>*emm*89 (12.7%)<br>*emm*3 (11.3%)<br>*emm*12 (8.2%)<br>*emm*28 (5.7%)	
2006-2009	National	All age group	4143	*emm*1[b]<br>*emm*59[b]<br>*emm*28[b]<br>*emm*3[b]<br>*emm*89[b]<br>*emm*12 [b]	Tyrrell et al, Clin Infect Dis, 2010

[a]In this work two distinct geographic areas were analyzed with significantly differences in their *emm* types distribution and frequencies, therefore the findings obtained by each specific area were separated

[b]The order of frequency of the major *emm* types was indicated, although the relative percentages of each *emm* type were not clearly indicated.
